# The *HFE* p.H63D (p.His63Asp) Polymorphism Is a Modifier of ALS Outcome in Italian and French Patients with *SOD1* Mutations

**DOI:** 10.3390/biomedicines11030704

**Published:** 2023-02-24

**Authors:** Antonio Canosa, Andrea Calvo, Gabriele Mora, Cristina Moglia, Maura Brunetti, Marco Barberis, Giuseppe Borghero, Claudia Caponnetto, Francesca Trojsi, Rossella Spataro, Paolo Volanti, Isabella Laura Simone, Fabrizio Salvi, Francesco Ottavio Logullo, Nilo Riva, Lucio Tremolizzo, Fabio Giannini, Jessica Mandrioli, Raffaella Tanel, Maria Rita Murru, Paola Mandich, Francesca Luisa Conforti, Marcella Zollino, Mario Sabatelli, Claudia Tarlarini, Christian Lunetta, Letizia Mazzini, Sandra D’Alfonso, Nathalie Guy, Vincent Meininger, Pierre Clavelou, William Camu, Adriano Chiò, on behalf of ITALSGEN Consortium

**Affiliations:** 1 ALS Centre, “Rita Levi Montalcini” Department of Neuroscience, University of Turin, 10126 Turin, Italy; 2 SC Neurologia 1U, Azienda Ospedaliero-Universitaria Città della Salute e della Scienza di Torino, 10126 Turin, Italy; 3 Institute of Cognitive Sciences and Technologies, National Research Council, 00185 Rome, Italy; 4 Neuroscience Institute of Turin (NIT), Regione Gonzole 10, 10043 Turin, Italy; 5 SC Genetica Medica U, Azienda Ospedaliero-Universitaria Città della Salute e della Scienza di Torino, 10126 Turin, Italy; 6 Department of Neurology, Azienda Ospedaliero-Universitaria di Cagliari and University of Cagliari, 09123 Cagliari, Italy; 7 Department of Neurosciences, Rehabilitation, Ophthalmology, Genetics, Maternal, and Child Health, University of Genoa, 16132 Genoa, Italy; 8 IRCCS Ospedale Policlinico San Martino, 16132 Genoa, Italy; 9 Department of Advanced Medical and Surgical Sciences, MRI Research Center SUN-FISM, University of Campania “Luigi Vanvitelli”, 80138 Naples, Italy; 10 ALS Clinical Research Centre, Department of Biomedicine, Neurosciences and Advanced Diagnostics, University of Palermo, 90129 Palermo, Italy; 11 Intensive Neurorehabilitation Unit, ALS Centre, IRCCS Istituti Clinici Scientifici Maugeri, 98073 Mistretta, Italy; 12 Neurological ALS Tertiary Centre, Department of Basic Medical Sciences, Neurosciences and Sense Organs, University of Bari, 70124 Bari, Italy; 13 “Il Bene” Centre for Immunological and Rare Neurological Diseases at Bellaria Hospital, IRCCS, Istituto Delle Scienze Neurologiche, 40125 Bologna, Italy; 14 Azienda Ospedaliera Ospedali Riuniti Marche Nord, Presidio di Fano, UOC Neurologia, 61032 Fano, Italy; 15 Fondazione IRCCS Istituto Neurologico Carlo Besta, SC Neurologia 3–Neuroalgologia, 20133 Milano, Italy; 16 Neurology Unit, ALS Clinic, San Gerardo Hospital and University of Milano-Bicocca, 20900 Monza, Italy; 17 Department of Medical, Surgical and Neurological Sciences, University of Siena, 53100 Siena, Italy; 18 Neurology Unit, Department of Neuroscience, S. Agostino Estense Hospital, Azienda Ospedaliero Universitaria di Modena, 41125 Modena, Italy; 19 Centre for Neuroscience and Neurotechnology (CfNN), Department of Biomedical, Metabolic and Neural Sciences, University of Modena and Reggio Emilia, 41124 Modena, Italy; 20 Division of Neurology, Department of Neurosciences, Santa Chiara Hospital, 38122 Trento, Italy; 21 Department of Medical Sciences, Multiple Sclerosis Centre, University of Cagliari, 09123 Cagliari, Italy; 22 Department of Pharmacy, Health and Nutritional Sciences, University of Calabria, 87036 Rende, Italy; 23 Institute of Genomic Medicine, Catholic University School of Medicine, 00168 Rome, Italy; 24 Medical Genetics, Policlinico A. Gemelli Foundation, IRCCS, 00168 Rome, Italy; 25 Department of Geriatrics, Neurosciences and Orthopedics, Clinic Center NEMO-Roma, Institute of Neurology, Catholic University School of Medicine, 00168 Rome, Italy; 26 Neurology, Policlinico A. Gemelli Foundation, IRCCS, 00168 Rome, Italy; 27 Neuromuscular Omnicentre (NEMO)-Fondazione Serena Onlus, 20162 Milan, Italy; 28 Istituti Clinici Scientifici Maugeri, IRCCS, 20138 Milan, Italy; 29 ALS Centre, Department of Neurology, Azienda Ospedaliera Universitaria Maggiore della Carità, 28100 Novara, Italy; 30 Department of Health Sciences, Interdisciplinary Research Center of Autoimmune Diseases, School of Medicine, University of Eastern Piedmont Amedeo Avogadro, 28100 Novara, Italy; 31 CRCSLA et Maladie du Neurone Moteur, Service de Neurologie, CHU de Clermont-Ferrand Clermont-Ferrand CEDEX 9 and UMR1107, Neurodol, UCA, 63000 Clermont-Ferrand, France; 32 Hôpital des Peupliers, Ramsay Générale de Santé, 75013 Paris, France; 33 Centre de Référence SLA, CHU and Université de Montpellier, 34295 Montpellier, France

**Keywords:** amyotrophic lateral sclerosis, *SOD1*, *HFE*, p.H63D, survival

## Abstract

*Background*: Data from published studies about the effect of HFE polymorphisms on ALS risk, phenotype, and survival are still inconclusive. We aimed at evaluating whether the p.H63D polymorphism is a modifier of phenotype and survival in *SOD1*-mutated patients. *Methods*: We included 183 *SOD1*-mutated ALS patients. Mutations were classified as severe or mild according to the median survival of the study population. Patients were screened for the *HFE* p.H63D polymorphism. Survival was calculated using the Kaplan–Meier modeling, and differences were measured by the log-rank test. Multivariable analysis was performed with the Cox proportional hazards model (stepwise backward). *Results*: *SOD1* severe mutation carriers show more frequent familial history for ALS and shorter survival compared to mild mutation carriers. Carriers and non-carriers of the p.H63D polymorphism did not differ in terms of sex ratio, frequency of positive familial history, age at onset, and bulbar/spinal ratio. In univariate and in Cox multivariable analysis using sex, age at onset, site of onset, family history, country of origin, and mutation severity as covariates, p.H63D carriers had a longer survival (*p* = 0.034 and *p* = 0.004). *Conclusions*: We found that *SOD1*-mutated ALS patients carrying the p.H63D HFE polymorphism have a longer survival compared to non-carriers, independently of sex, age and site of onset, family history, nation of origin, and severity of mutations, suggesting a possible role as disease progression modifier for the p.H63D *HFE* polymorphism in *SOD1*-ALS.

## 1. Introduction

Amyotrophic lateral sclerosis (ALS) is a neurodegenerative disease affecting upper and lower motor neurons leading to death approximately three years after the onset, usually from respiratory failure. Genetic mutations are detectable in about two thirds of familial cases (fALS) and more than 10% of apparently sporadic patients, with *C9ORF72*, *SOD1*, *TARDBP,* and *FUS* being the most frequent causative genes [1]. Furthermore, different genes have been identified as modifiers of disease survival. Polymorphisms of *UNC13A* [2,3] and *NIPA1* [4] genes and intermediate-length expansion of the *ATXN2* [5] gene have been associated with reduced survival. The impact of *HFE* gene variants on ALS risk and phenotype has been investigated as it is involved in iron homeostasis, and the p.C282Y and p.H63D polymorphisms are associated with hereditary hemochromatosis (HH). HH is characterized by iron accumulation in the liver, adrenal glands, heart, skin, gonads, joints, and pancreas. Indeed, there are data suggesting that brain iron overload, possibly related to oxidative stress, has a role in different neurodegenerative diseases, including Alzheimer’s disease and Parkinson’s disease [6]. The possible role of the p.H63D (p.His63Asp) polymorphism of the *HFE* gene as a risk factor for ALS has been evaluated in several studies with conflicting results. Of note are two recent meta-analyses that do not show any detrimental effect on disease risk [7,8].

In a large series of Italian ALS patients, we previously found that this polymorphism did not influence ALS phenotype and survival, with the possible exception of patients carrying *SOD1* mutations [9]. Nevertheless, the number of *SOD1* patients in that series was relatively small (*n* = 26). The aim of the present study is to assess whether the *HFE* p.H63D (rs1799945) common polymorphism is a modifier of phenotype and survival in a series of Italian and French *SOD1*-mutated patients, in order to replicate the results of the previous study in a larger cohort.

## 2. Materials and Methods

### 2.1. Patients

We included ALS patients carrying *SOD1* mutations, collected through the Italian ALS Genetic (ITALSGEN) consortium and at three French ALS centres (Montpellier, Clermont-Ferrand, Paris) in the period 1996–2015. All patients were of Caucasian ethnicity and did not carry mutations in the other most common ALS-related genes (*C9ORF72*, *TARDBP*, and *FUS*). In order to control for the heterogeneity of clinical course related to different *SOD1* mutations, each mutation was classified either as severe or mild according to the median survival of the whole study population (i.e., 7.41 years). Accordingly, mutations whose median survival was <7.41 years were considered severe and those whose median survival was >7.41 years were considered mild. A further sensitivity analysis was performed excluding all mutations identified in only one or two patients.

### 2.2. Genotyping

All the coding exons and 50 bp of the flanking intron–exon boundaries of *SOD1* were PCR-amplified, sequenced using the BigDye Terminator v3.1 sequencing kit (Applied Biosystems Inc.), and run on an ABIPrism 3500 genetic analyzer. The exon 2 of *HFE* was amplified by PCR and analyzed by denaturing high-performance liquid chromatography (DHPLC) (Transgenomic, Inc., Omaha, NE, USA). PCR products with heteroduplex profiles were sequenced on an ABI 3500 sequencer (Life Technologies, Foster City, CA, USA) with BigDye termination v.1.1 (Life Technologies) technologies according to standard protocols. Samples with homozygous profiles were coupled with a wild-type reference, denaturated and re-analyzed by DHPLC in order to also detect homozygous sequence alterations. If a mixed profile was positive, the original sample was sequenced. All sequencing products were analyzed with SeqScape Software v.3.0 (Applied Biosystems—Life Technologies). Other ALS-related genes were screened according to previously described procedures [10].

### 2.3. Statistical Analysis

The adherence to the Hardy–Weinberg equilibrium was tested for the *HFE* alleles through the appropriate equation. Comparisons between means were made with the Student’s *t*-test or analysis of variance; comparison between categorical variables was made with the χ^2^ test and Fisher’s test when applicable; Levene’s test was used to confirm the equality of variances. Survival was calculated from onset to death/tracheostomy or censoring date (30 June 2016) using the Kaplan–Meier survival modeling, and differences in survival were measured by the log-rank test. No patients were lost to follow up. Multivariable analysis was performed with the Cox proportional hazards model (stepwise backward) with a retention criterion of *p* < 0.1. The significance level was set at *p* < 0.05. Data were processed using SPSS statistical package, version 22.0 (IBM Corporation, Chicago, IL, USA).

## 3. Results

A total of 183 Italian and French ALS patients carrying different mutations of *SOD1* gene were included (30 from France and 153 from Italy). *SOD1* mutations found in our sample are reported with the relative frequency (in decreasing order) and classification (mild/severe) in Table 1.

Table 2 shows demographic and clinical characteristics of patients of the whole series and of the two groups (mild and severe mutation carriers).

Familial history for ALS is significantly more reported in patients with severe mutations compared to those carrying mild mutations (*p* = 0.04), possibly due to higher penetrance of severe mutations compared to mild mutations. A significant difference is found in median survival, as expected, based on the criterion used to classify mutations as mild or severe.

Patients were assessed for the rs1799945 (p.H63D) polymorphism of the *HFE* gene. The following allelic frequencies were found: CC (absence of the p.H63D polymorphism), 127 cases (69.4%); CG (heterozygous for p.H63D), 51 cases (27.9%); GG (homozygous for p.H63D), 5 cases (2.7%). The frequencies of observed *HFE* genotypes meet the Hardy–Weinberg equilibrium (Table 3).

Patient groups corresponding to different *HFE* genotypes did not differ in terms of sex ratio (*p* = 0.165), frequency of familial history (*p* = 0.863), and age at onset (*p* = 0.904). The bulbar/spinal ratio resulted different (*p* < 0.01) but the difference becomes not significant considering together heterozygous and homozygous carriers of the p.H63D polymorphism.

**Figure 1:**
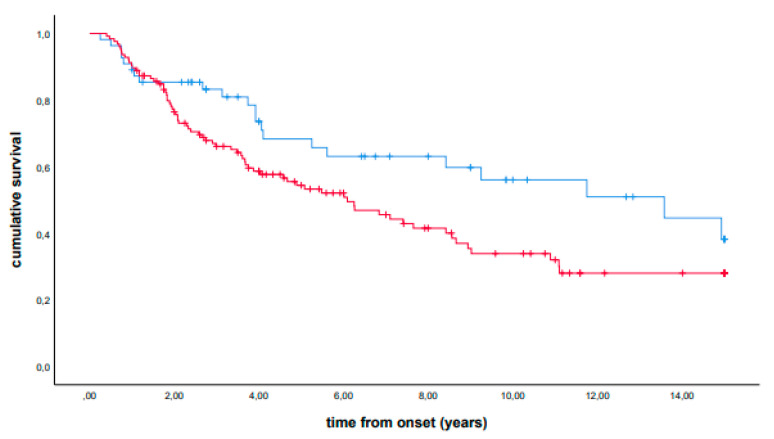
Cumulative survival (Kaplan–Meier curves) of patients carrying the p.H63D (CG + GG) polymorphism (blue line) versus non-carrier patients (red line).

In univariate analysis, patients carrying the p.H63D (CG + GG) polymorphism showed a longer median survival (median survival, 13.58 years, IQR 3.92-NA, vs 6.09 years, IQR 2.08-NA, *p* = 0.034). A stratified analysis showed that the observed effect was confirmed in each group (for mild and severe mutation carriers, see Supplemental Figures S1 and S2, respectively). The presence of the p.H63D polymorphism also remained significant in Cox multivariable analysis using sex, age at onset, site of onset, family history, country of origin, and severity of mutations as covariates (hazard ratio, 0.49, 95% CI 0.30–0.80, *p* = 0.004). Kaplan–Meier curves are reported in Figure 1. Similar results were obtained excluding mutations identified in only one or two patients (data not reported).

## 4. Discussion

Among 183 Italian and French ALS patients carrying *SOD1* mutations, we found that the presence of the G allele of the *HFE* gene (i.e., p.H63D polymorphism) was significantly associated with a longer survival. Published data about *HFE* variants and ALS risk, phenotype, and survival are conflicting. Some studies reported a higher frequency of the p.H63D polymorphism in ALS patients compared to healthy controls, but without any influence on male/female ratio, age at and site of onset, and survival [11,12,13,14]. Sutedja et al. reported a positive association between homozygosity for the p.H63D polymorphism and ALS, suggesting that *HFE* is a contributing factor in the development of the disease in the Dutch population. In addition, they found that heterozygosity was associated with a higher age at ALS onset, possibly indicating that p.H63D is a risk factor for a later-onset form of ALS [15]. Other studies failed to detect any association between the p.H63D variant and ALS risk, phenotype, and survival [16,17]. Particularly, a survey collecting 3962 ALS patients and 5072 healthy controls showed no difference in the p.H63D allele frequency between patients and controls, and no effect of the p.H63D polymorphism on age at onset and survival [7]. Furthermore, a metanalysis published in 2014 did not observe any evidence for an association of the p.H63D polymorphism with ALS risk [8]. Interestingly, a study by Su and colleagues [18] reported that the p.H63D *HFE* polymorphism (either homozygous or heterozygous) was associated with increased disease duration and decreased muscle superoxide dismutase-1 expression in ALS patients. However, the sample size was small (22 ALS patients with wild-type *HFE* and 16 ALS patients either homozygous or heterozygous for the p.H63D *HFE* polymorphism) and a selection bias was contemplated by the authors, since many of the patients selected for muscle biopsy underwent this procedure because their clinical and/or electrodiagnostic findings were in some way atypical. In 2015, a study [9] including 1119 Italian and 232 Sardinian ALS patients and 1302 Italian and 121 Sardinian age- and gender-matched healthy controls did not find any significant difference in either population. Patients with CC, CG, and GG genotypes did not differ by age at onset and site of onset. No difference of survival was found considering both the CC/CG/GG phenotypes and the presence of a G allele in either cohorts of patients. In the 26 patients with *SOD1* mutations, an increased survival was found in patients with CG or GG compared with CC genotype or in patients carrying the G allele (dominant assumption) (*p* = 0.04). This finding was confirmed by the multivariable Cox model, where the G allele was retained as an independent prognostic factor (*p* = 0.03). The conflicting findings of published studies may arise from different aspects. First, some papers are based on small, underpowered series; second, in some studies, controls are not matched by ethnic origin to cases. To overcome the limitation of the small sample size, in the present study we enrolled 183 Italian and French ALS patients carrying *SOD1* mutations and showed that the presence of the G allele of the *HFE* gene (i.e., p.H63D polymorphism) was significantly associated with a longer survival. Conversely, in the *SOD1* transgenic mouse, the p.His67Asp polymorphism of the *HFE* gene (analogue of the p.His63Asp human polymorphism) has been observed to negatively influence the survival of the *SOD1* transgenic mouse [19]. The double transgenic mice had shorter survival and disease duration compared to *SOD1* mice, but only in the female group. No further differences between the two transgenic mice were found in age at onset, motor neuron loss, and total iron concentrations in lumbar spinal cord. Such conflicting data suggest the possibility that genetic interactions in mice are biologically different compared to humans. The mechanism through which the p.His63Asp *HFE* polymorphism might prolong the survival of *SOD1*-mutated ALS patients remains to be elucidated. Although some *HFE* variants are related to HH, they are common in the Caucasian population, probably because they provide some advantage to asymptomatic carriers. Recent studies suggest that *HFE* variants, including the p.H63D, can positively influence the immune system, the general fitness, and reproductive status of carriers [20]. The increase in iron uptake may have helped humans, particularly women of reproductive age, to compensate for the reduction in iron content in the cereal grain-based diet that took the place of the paleo-diet (rich in red meat) in Europe in the Neolithic Age, when the first *HFE* mutation arose [21]. We can hypothesize that the p.H63D polymorphism of *HFE* may provide some advantage to carriers in the presence of a pathogenic *SOD1* mutation. Nevertheless, such a hypothesis needs to be explored by further studies. The possible involvement of iron homeostasis dysregulation in ALS pathogenesis deserves further evaluation since it could be a therapeutic target. Currently, a phase 2–3, multicentre, parallel-group, placebo-controlled, randomised clinical trial of deferiprone, an iron chelator, as disease-modifying strategy is currently ongoing (NCT03293069). Nevertheless, the precise comprehension of the characteristics of the eventual dysregulation of iron metabolism in ALS is of outstanding importance to understand how to modulate it through therapeutic interventions.

## 5. Conclusions

We found that *SOD1*-mutated ALS patients carrying the p.H63D polymorphism of *HFE* have a significantly longer median survival compared to non-carriers, and the result is independent of sex, age at onset, site of onset, positive family history, nation of origin, and severity of mutations. The underlying basis of such an effect of the *HFE* genotype on *SOD1* ALS survival needs further investigation to be clarified.

## Figures and Tables

**Table 1:** List of *SOD1* mutations in order of decreasing frequency in our series (*n* = 183).

Mutation	Number of Carriers	Percentage	Mutation	Number of Carriers	Percentage
p.Gly94Asp	25	13.7	p.Ile150Thr	5	2.7
p.Asp91Ala (heterozygous)	14	7.7	p.Gly94Cys	4	2.2
p.Leu145Phe	14	7.7	p.Ser135Asn †	4	2.2
p.Leu85Phe †	11	6.0	p.Thr138Ala	3	1.6
p.Gly42Ser †	10	5.5	p.Gly11Arg	2	1.1
p.Ala5Val †	9	4.9	p.Ala96Gly	2	1.1
p.Asn66Ser	7	3.8	p.Asp110Tyr	2	1.1
p.Asp12Tyr	6	3.3	p.Ile114Thr	2	1.1
p.Glu134del	6	3.3	p.Asp125Gly	2	1.1
p.Asn20Ser	5	2.7	p.Gly148Asp	2	1.1
p.Gly73Ser †	5	2.7	p.Gly148Ser	2	1.1
p.Asp91Ala (homozygous)	5	2.7	Others ‡	36	19.7

Severe mutations are labelled with the symbol “†”. The nomenclature used is in agreement with the recommendations for the description of sequence variants of the Human Genome Variation Society (http://varnomen.hgvs.org/, accessed on 29 Debember 2022). ‡ Others (mutations carried by one subject): p.Ala5Thr, p.Val6Met,† p.Phe21Leu, p.Glu22Gly, p.Gln23Arg,† p.Leu39Arg,† p.Leu39Val,† p.Phe46Cys, p.His47Arg, p.His49Arg, p.Ser60Arg, p.Ser60Ile,† p.Asn66Thr,† p.Leu68Pro,† p.His81Arg, p.Gly86Ser,† p.Val88Met, p.Asp91Asn,† p.Ala96Thr, p.Glu101Lys, p.Asp102Gly,† p.Ile106Phe, p.Leu107Phe,† p.Ser108LeufsTer15, p.Ile113Met,† p.Arg116Gly, p.Val119Met, p.Val120Leu, p.Glu122Gly, p.Lys129del, p.Ser135Gly, p.Asn140Asp, p.Gly143Glu, p.Ala146Thr, p.Gly148Ala, p.Gly148Cys.

**Table 2:** Demographic and clinical characteristics of the whole series (*n* = 183) and of the two groups (mild and severe mutation carriers).

	Whole Series	Mild Mutation Carriers	Severe Mutation Carriers	*p*
Sex *Female (%)* *Male (%)*	98 (53.6%) 85 (46.4%)	75 (56.8%) 57 (43.2%)	23 (45.1%) 28 (54.9%)	*p* = 0.15
Familial Status *FALS (%)* *SALS (%)*	111 (60.7%) 72 (39.3%)	74 (56.1%) 58 (43.9%)	37 (72.5%) 14 (27.5%)	*p* = 0.04
Age at onset, mean [SD]	53.7 [12.1]	53.6 [12.1]	53.8 [12.2]	*p* = 0.93
Site of onset *Bulbar (%)* *Spinal (%)*	10 (5.5%) 173 (94.5%)	8 (6.1%) 124 (93.9%)	2 (3.9%) 49 (96.1%)	*p* = 0.57
Survival (years), median [IQR]	7.41 [2.58-NA]	9.02 [4.06-NA]	1.99 [1–4.03]	*p* < 0.001
Total	183	132	51	

*p*-values refer to the comparison between mild and severe mutation carriers (when significant, they are reported in bold). FALS: familial ALS. SALS: sporadic ALS. SD: standard deviation; IQR: interquartile range.

**Table 3:** Calculation of Hardy–Weinberg equilibrium for *HFE* genotypes.

HFE Genotypes	Observed	Expected
CC	127	127.1
CG	51	50.8
GG	5	5.1
Minor allele frequency	0.17	
χ^2^ test *p*-value	0.96 with 1 degree of freedom	

## Data Availability

Data are available upon request from interested researchers.

## Supplementary Materials

The following supporting information can be downloaded at: https://www.mdpi.com/article/10.3390/biomedicines11030704/s1: Supplemental Figure S1 [Univariate analysis in the mild mutation carrier group: Kaplan–Meier curves showing that patients carrying the p.H63D (CG + GG) polymorphism (blue line) have a longer median survival compared to non-carriers (red line)]; Supplemental Figure S2 [Univariate analysis in the severe mutation carrier group: Kaplan–Meier curves showing that patients carrying the p.H63D (CG + GG) polymorphism (blue line) have a longer median survival as compared to non-carriers (red line)]; list of the members of the ITALSGEN consortium.
