# Supplementary material for: The HFE p.H63D (p.His63Asp) Polymorphism Is a Modifier of ALS Outcome in Italian and French Patients with SOD1 Mutations
Source: Biomedicines. 2023 Feb 24;11(3):704. doi: 10.3390/biomedicines11030704 (PMC10044845; doi:10.3390/biomedicines11030704)

Members of the ITALSGEN Consortium

Stefania M. Angelocola (Neurology Unit, AV4, ASUR Marche, Fermo, Italy); Francesco P. Ausiello (Department of Neurosciences, Reproductive Sciences and Odontostomatology, University of Naples Federico II, Naples, Italy); Ilaria Bartolomei (Center for Diagnosis and Cure of Rare Diseases, Department of Neurology, IRCCS Institute of Neurological Sciences, Bologna, Italy); Stefania Battistini (Department of Medical, Surgical and Neurological Sciences, University of Siena, Siena, Italy); Enrica Bersano (3rd Neurology Unit, Motor Neuron Diseases Centre Fondazione IRCCS, Istituto Neurologico Carlo Besta, Milano, Italy); Giulia Bisogni (NeuroMuscular Omnicentre, NEMO, Serena Onlus Foundation - Policlinico A. Gemelli, Roma, Italy); Corrado Cabona (Department of Neurosciences, Ophthalmology, Genetics, Rehabilitation, Maternal and Child Health, IRCCS Ospedale Policlinico San Martino, Genova, Italy); Fabrizio Canale, Department of Advanced Medical and Surgical Sciences University of Campania "Luigi Vanvitelli", Naples, Italy); Teresa A. Cantisani (Struttura complessa di Neurofisiopatologia, Azienda Ospedaliera di Perugia, Perugia, Italy); Margherita Capasso (Unit of Neurology, Ospedale Clinicizzato SS Annunziata, Chieti, Italy); Patrizio Cardinali, Neurology Unit, AV4, ASUR Marche, Fermo, Italy); Paola Carrera (Department of Neurology and Institute of Experimental Neurology, INSPE, IRCCS San Raffaele Scientific Institute, Milano, Italy); Federico Casale ("Rita Levi Montalcini" Department of Neuroscience, Amyotrophic Lateral Sclerosis Centre, University of Torino, Torino, Italy); Tiziana Colletti (ALS Clinical Research Centre, Bi.N.D., University of Palermo, Palermo, Italy); Amelia Conte (NeuroMuscular Omnicentre, NEMO, Serena Onlus Foundation - Policlinico A. Gemelli, Roma, Italy); Elisa Conti (Neurology Unit, "San Gerardo" Hospital; Monza, Italy; School of Medicine and Surgery and Milan Center for Neuroscience, NeuroMI, University of Milano-Bicocca, Milano, Italy); Massimo Corbo (Department of Neurorehabilitation Sciences, Casa Cura Policlinico, Milano, Italy); Stefania Cuccu (Neurology Unit, Monserrato University Hospital, Cagliari, University of Cagliari, Italy); Eleonora Dalla Bella

(3rd Neurology Unit, Motor Neuron Diseases Centre, Fondazione IRCCS, Istituto Neurologico Carlo Besta, Milano, Italy); Eustachio D'Errico (Department of Medical Sciences and Neuroscience, University “Aldo Moro” of Bari, Policlinico, Bari, Italy); Giovanni De Marco (“Rita Levi Montalcini” Department of Neuroscience, Amyotrophic Lateral Sclerosis Centre, Torino, Italy); Raffaele Dubbioso (Department of Neurosciences, Reproductive Sciences and Odontostomatology, University of Naples Federico II, Naples, Italy); Carlo Ferrarese (Neurology Unit, "San Gerardo" Hospital, Monza, Italy; School of Medicine and Surgery and Milan Center for Neuroscience, NeuroMI, University of Milano-Bicocca, Milano, Italy); Pilar M. Ferraro (Department of Neurosciences, Ophthalmology, Genetics, Rehabilitation, Maternal and Child Health, IRCCS Ospedale Policlinico San Martino, Genova, Italy); Massimo Filippi (Department of Neurology, IRCCS ”San Raffaele Scientific Institute”, Milano, Italy; Neuroimaging Research Unit, Division of Neuroscience, Vita-Salute San Raffaele University, San Raffaele Scientific Institute, Milano, Italy); Nicola Fini (Department of Neurosciences, Ospedale Civile S. Agostino Estense, Azienda Ospedaliero Universitaria di Modena, Modena, Italy); Gianluca Floris (Neurology Unit, Monserrato University Hospital, University of Cagliari, Cagliari, Italy); Giuseppe Fuda (“Rita Levi Montalcini' Department of Neuroscience, Amyotrophic Lateral Sclerosis Centre, University of Torino, Torino, Italy); Salvatore Gallone (“Rita Levi Montalcini” Department of Neuroscience, Amyotrophic Lateral Sclerosis Centre, University of Torino, Torino, Italy) Giulia Gianferrari (Department of Biomedical, Metabolic and Neural Sciences, University of Modena and Reggio Emilia, 41124 Modena, Italy.); Maurizio Grassano (“Rita Levi Montalcini” Department of Neuroscience, Amyotrophic Lateral Sclerosis Centre, University of Torino, Torino, Italy); Lucia Greco (NEMO Clinical Center Milano, Fondazione Serena Onlus, Milano, Italy); Carlo Guidi (Neurology Unit of Forlì, AUSL Romagna, Forlì, Italy); Barbara Iazzolino (“Rita Levi Montalcini' Department of Neuroscience, Amyotrophic Lateral Sclerosis Centre, University of Torino, Torino, Italy); Alessandro Introna (Department of Medical Sciences and Neuroscience, University “Aldo Moro” of Bari, Policlinico, Bari, Italy); Vincenzo La Bella (ALS Clinical Research Centre, Bi.N.D.,

University of Palermo, Palermo, Italy); Serena Lattante (Genetica Medica; Fondazione Policlinico Universitario A. Gemelli IRCCS, Roma, Italy; Dipartimento Universitario Scienze della Vita e Sanità Pubblica, Sezione di Medicina Genomica, Università Cattolica del Sacro Cuore Facoltà di Medicina e Chirurgia, Roma, Italy); Giuseppe Lauria (3rd Neurology Unit, Motor Neuron Diseases Centre, Fondazione IRCCS, Istituto neurologico Carlo Besta, Milano, Italy; Department of Biomedical and Clinical Sciences Luigi Sacco, University of Milan, Milano, Italy); Rocco Liguori (Center for Diagnosis and Cure of Rare Diseases, Department of Neurology, University of Bologna, Bologna, Italy); Giancarlo Logroscino (Department of Basic Medical Sciences, Neuroscience and Sense Organs, University of Bari "Aldo Moro", Bari, Italy; Center for Neurodegenerative Diseases and the Aging Brain, Department of Clinical Research in Neurology, Pia Fondazione Cardinale G. Panico, Tricase, Italy); Francesca Lupidi (Neurological Clinic, AOU Ospedali Riuniti of Ancona, Ancona, Italy); Umberto Manera ("Rita Levi Montalcini" Department of Neuroscience, Amyotrophic Lateral Sclerosis Centre, University of Torino, Torino, Italy); Fiore Manganelli (Department of Neurosciences, Reproductive Sciences and Odontostomatology, University of Naples Federico II, Napoli, Italy); Giuseppe Marangi (Genetica Medica, Fondazione Policlinico Universitario A. Gemelli IRCCS, Roma, Italy; Dipartimento Universitario Scienze della Vita e Sanità Pubblica, Sezione di Medicina Genomica, Università Cattolica del Sacro Cuore Facoltà di Medicina e Chirurgia, Roma, Italy); Kalliopi Marinou (Department of Neurorehabilitation, Istituti Clinici Scientifici Maugeri IRCCS, Institute of Milan, Milano, Italy); Maria Giovanna Marrosu (Department of Neurology, Azienda Universitario Ospedaliera di Cagliari and University of Cagliari, Cagliari, Italy); Ilaria Martinelli (Department of Neurosciences, Ospedale Civile S. Agostino Estense, Azienda Ospedaliero Universitaria di Modena, Modena, Italy); Sonia Messina (NEuroMuscular Omnicentre NEMO Sud, Fondazione Aurora, OUC Neurology and Neuromuscular Disorders, University of Messina, Messina, Italy); Maria Rosaria Monsurrò (Department of Advanced Medical and Surgical Sciences, University of Campania "Luigi Vanvitelli", Napoli, Italy); Lorena Mosca (Department of Laboratory Medicine, Medical Genetics,

Niguarda Ca' Granda Hospital, Milano, Italy); Paola Origone (Department of Neurosciences, Ophthalmology, Genetics, Rehabilitation, Maternal and Child Health, IRCCS Ospedale Policlinico San Martino, Genova, Italy); Enzo Ortu (Neurology Unit, ASSL Sassari, Sassari, Italy); Carla Passaniti (Department of Advanced Medical and Surgical Sciences, University of Campania "Luigi Vanvitelli", Napoli, Italy); Cristina Petrelli (Neurology Unit, AV3, ASUR Marche, Macerata, Italy); Antonio Petrucci (Neurology Department, San Camillo Hospital, Roma, Italy); Angelo Pirisi (Department of Biomedical and Surgical Sciences, Section of Neurological, Psychiatric and Psychological Sciences, University of Ferrara, Ferrara, Italy); Susanna Pozzi (NEMO Clinical Center Milano, Fondazione Serena Onlus, Milano, Italy); Maura Pugliatti (Department of Biomedical and Surgical Sciences, Section of Neurological, Psychiatric and Psychological Sciences, University of Ferrara, Ferrara, Italy); Angelo Quattrini (Department of Neurology, IRCCS ”San Raffaele Scientific Institute”, Milano, Italy); Claudia Ricci (Department of Medical, Surgical and Neurological Sciences, University of Siena, Siena, Italy); Giulia Riolo (Department of Medical, Surgical and Neurological Sciences, University of Siena, Siena, Italy); Massimo Russo (OUC Neurology and Neuromuscular Disorders, University of Messina, Messina, Italy); Paolina Salamone (“Rita Levi Montalcini” Department of Neuroscience, Amyotrophic Lateral Sclerosis Centre, University of Torino, Torino, Italy); Marco Salivetto (NEMO Clinical Center Milano, Fondazione Serena Onlus, Milano, Italy); Marialuisa Santarelli (Department of Medicine, Azienda Complesso Ospedaliero, San Filippo Neri, Roma, Italy); Luca Sbaiz (Department of Medical Genetics, Azienda Ospedaliero Universitaria Città della Salute e della Scienza, Torino, Italy); Riccardo Sideri (Department of Neurorehabilitation, Istituti Clinici Scientifici Maugeri IRCCS, Institute of Milan, Milano, Italy); Cecilia Simonini (Department of Neurosciences, Ospedale Civile S. Agostino Estense, Azienda Ospedaliero Universitaria di Modena, Modena, Italy); Gioacchino Tedeschi (Department of Advanced Medical and Surgical Sciences, University of Campania "Luigi Vanvitelli", Napoli, Italy); Anna Ticca (Department of Neurology, Azienda Ospedaliera San Francesco, Nuoro, Italy); Antonella Torriello (ALS Center, Operative Unit of

Neurology, AOU "San Giovanni di Dio e Ruggi d'Aragona", Salerno, Italy); Stefania Tranquilli (Neurologic Unit, Monserrato University Hospital, University of Cagliari, Cagliari, Italy); Rosario Vasta ("Rita Levi Montalcini" Department of Neuroscience, Amyotrophic Lateral Sclerosis Centre, University of Torino, Torino, Italy); Veria Vacchiano (Center for Diagnosis and Cure of Rare Diseases, Department of Neurology, IRCCS Institute of Neurological Sciences, Bologna, Italy); Giuseppe Vita (OUC Neurology and Neuromuscular Disorders, University of Messina, Messina, Italy); Elisabetta Zucchi (Department of Biomedical, Metabolic and Neural Sciences, University of Modena and Reggio Emilia, 41124 Modena, Italy.).

Supplemental Figure S1. Univariate analysis in the mild mutation carrier group: Kaplan-Meier curves showing that patients carrying the p.H63D (CG + GG) polymorphism (blue line) had a longer median survival as compared to non-carriers (red line).

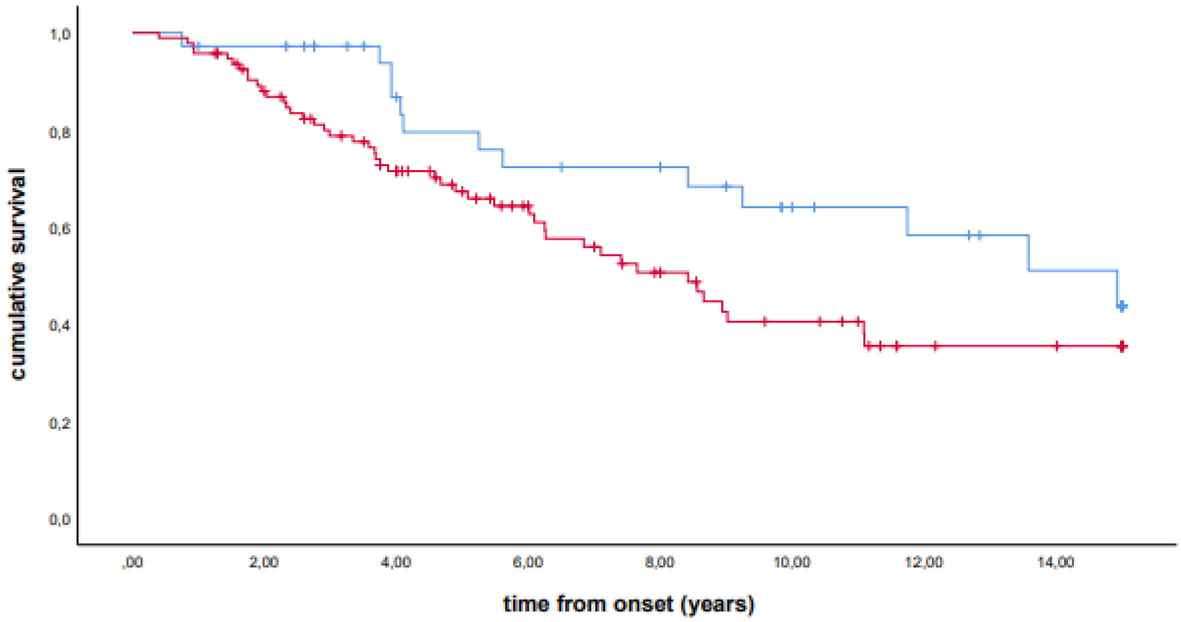

Supplemental Figure S2. Univariate analysis in the severe mutation carrier group: Kaplan-Meier curves showing that patients carrying the p.H63D (CG + GG) polymorphism (blue line) had a longer median survival as compared to non-carriers (red line).

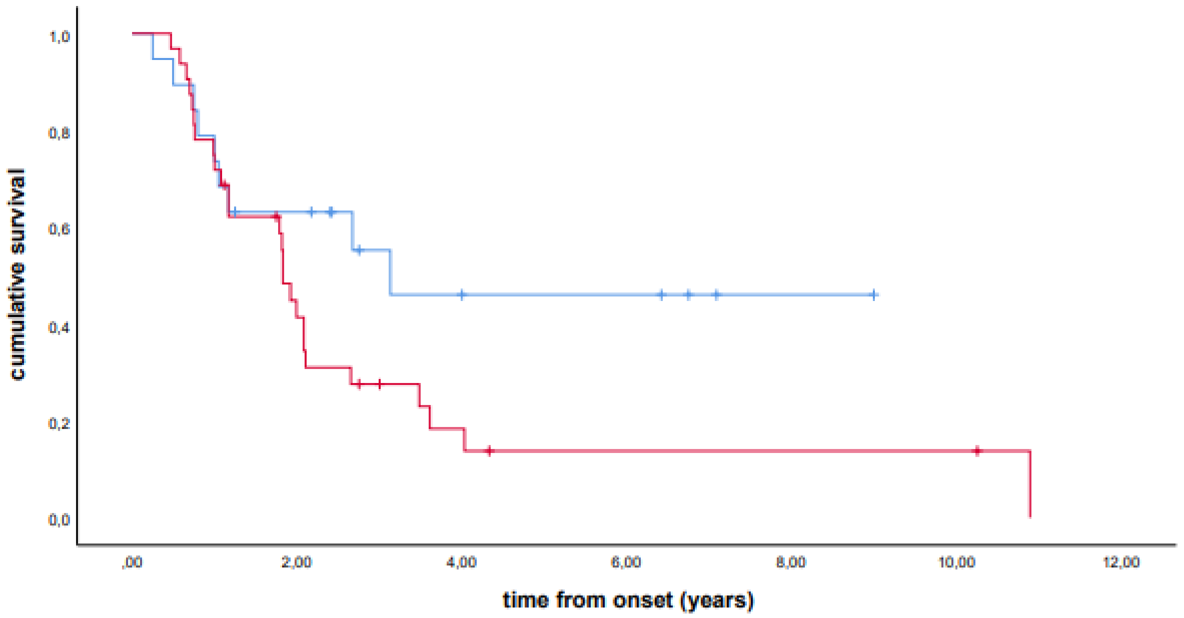

Supplement: Supplementary file 1 [file biomedicines-11-00704-s001.zip › biomedicines-2165113-supplementary.pdf]
